# Comparison of early clinical outcomes between CR and PS prostheses in total knee arthroplasty for rheumatoid arthritis patients - a retrospective cross-sectional study

**DOI:** 10.3389/fsurg.2025.1522588

**Published:** 2025-05-06

**Authors:** Haoyuan Ding, Dapeng Han, Nanshan Ma, Chenxin Gao, Jie Yao, Guilin Ouyang

**Affiliations:** ^1^ Shanghai University of Traditional Chinese Medicine, Shanghai, China; ^2^ Department of Orthopedic Surgery, Guanghua Hospital Affiliated to Shanghai University of Traditional Chinese Medicine, Shanghai, China

**Keywords:** rheumatoid arthritis, total knee arthroplasty, CR prosthesis, PS prosthesis, retrospective study

## Abstract

**Objective:**

To compare the early clinical outcomes of posterior cruciate ligament-retaining (CR) and posterior stabilized (PS) knee prostheses in total knee arthroplasty for patients with rheumatoid arthritis.

**Methods:**

A retrospective analysis was conducted on 74 patients with rheumatoid arthritis (RA) who underwent unilateral total knee arthroplasty (TKA) from January 2021 to December 2022. Among these, 39 patients received CR prostheses (CR group), while 35 received PS prostheses (PS group). Data on operation time, intraoperative blood loss, hospital stay, preoperative and postoperative Visual Analogue Scale (VAS) scores, American Knee Society Score (AKSS), Functional Joint Score-12 (FJS-12) scores, Health Assessment Questionnaire scores (HAQ) and postoperative complications were recorded and compared between the two groups.

**Results:**

All 74 patients successfully completed the surgery without complications. The average operation time for the CR group was shorter than that of the PS group, with no statistically significant differences in intraoperative blood loss or hospital stay. Both groups showed improved postoperative AKSS scores, VAS scores, and HAQ Scores compared to preoperative levels. Between-group comparisons showed no statistical differences in postoperative AKSS, VAS, HAQ scores. However, the CR group had significantly higher FJS-12 scores at 6 and 12 months postoperatively compared to the PS group.

**Conclusion:**

Both CR and PS prostheses can achieve good clinical outcomes in TKA for RA patients. Compared to PS prostheses, CR prostheses may provide better knee proprioception postoperatively, as indicated by higher FJS-12 scores at 6 and 12 months postoperatively.

## 1 Introduction

Rheumatoid arthritis (RA) is a systemic autoimmune disease characterized by chronic, symmetrical inflammation of multiple joints and extra-articular manifestations, with an estimated global incidence of about 0.2% (1). Knee joints, being the largest and most complex synovial joints, are frequently affected in RA. Patients with RA affecting the knee often suffer from pain and functional limitations due to synovial hyperplasia, cartilage degradation, and joint deformities. Total knee arthroplasty (TKA), a highly successful procedure, remains the most effective surgical option for RA patients when conservative treatments are ineffective (2, 3). Patients with rheumatoid arthritis often present with severe knee joint destruction and deformities. Additionally, the risk of postoperative knee joint infection and prosthesis loosening is significantly higher than in osteoarthritis, necessitating individualized surgical strategies and precise perioperative management.

Currently, TKA predominantly uses posterior cruciate ligament-retaining (CR) and posterior stabilized (PS) prostheses. Studies have demonstrated that both CR and PS prostheses can significantly improve knee function, alleviate pain, and boost quality of life (4). Given the scarcity of research on prosthesis selection in RA patients, this retrospective study aims to compare the early clinical outcomes of CR and PS prostheses following TKA for RA patients.

## 2 Materials and methods

### 2.1 General information

A retrospective analysis was conducted on RA patients who underwent TKA from January 2021 to December 2022. Patients were divided into CR and PS groups based on the type of knee prosthesis used, which was determined according to the surgeon's clinical judgment, considering factors such as preoperative ligament integrity, bone quality, joint deformity, and intraoperative stability assessment. This study did not employ randomization or blinding due to its retrospective design.

### 2.2 Inclusion and exclusion criteria

#### 2.2.1 Inclusion criteria

Diagnosed with RA according to the 2010 criteria of the American College of Rheumatology (ACR)/European League Against Rheumatism (EULAR) and categorized as Steinbrocker grade III or IV, with ineffective conservative treatment and clear surgical indications;

Patients undergoing unilateral knee surgery for the first time;

Varus or valgus deformity less than 15°;

No anesthesia contraindications, ASA classification of I or II, with signed informed consent.

#### 2.2.2 Exclusion criteria

Patients with severe knee instability requiring constrained prostheses or extended stems;

Patients with severe complications requiring reoperation;

Incomplete clinical and imaging follow-up data.

### 2.3 Perioperative management of antirheumatic medications

For patients receiving conventional synthetic disease-modifying antirheumatic drugs (csDMARDs) and glucocorticoids preoperatively, these medications should be continued perioperatively without dosage adjustments. For patients taking targeted synthetic disease-modifying antirheumatic drugs (tsDMARDs) before surgery, the medication should be discontinued at least three days prior to the procedure. For patients on biological disease-modifying antirheumatic drugs (bDMARDs), all biologics should be stopped preoperatively and resumed only after proper wound healing and confirmation of no infection at both surgical and non-surgical sites, typically around two weeks postoperatively (5).

### 2.4 Preoperative evaluation

All patients underwent a comprehensive physical examination and imaging assessments, including standing, lateral, and patellar views of the knee joint, as well as full-length lower limb x-rays and MRI scans. PCL injuries were classified into four grades based on MRI findings (6, 7):

Grade 0: No significant signal change in PCL; intact structure with no signs of congestion or edema around the ligament.

Grade 1: Increased signal on T1 and T2 sequences within the ligament, but intact fibers, with no changes in shape, thickness, or length, and damage area <1/2.

Grade 2: High signal changes on T1 and T2 sequences, with thickening, swelling, or hemorrhage, irregular or partially discontinuous fibers, damage area ≥1/2.

Grade 3: Significant signal enhancement on MRI, with ligament discontinuity, retraction, or wavy or clumped morphology, indicating complete rupture.

For patients with no PCL injury on physical examination and MRI grading of 0 or 1, CR prostheses were used if the PCL was intact during surgery; otherwise, PS prostheses were chosen. For those with PCL laxity and MRI grading of 2 or 3, PS prostheses were applied (8).

### 2.5 Follow-up and outcome measures

Follow-up was conducted preoperatively and at 1, 6, and 12 months postoperatively. Outcome measures included AKSS score, VAS score, HAQ score, FJS-12 score, and any postoperative complications. AKSS is a comprehensive knee evaluation scale proposed by the American Knee Society in 1989, which includes knee pain scores (Knee Score) and functional ability scores (Function Score). VAS is widely used for pain assessment, reflecting subjective pain severity. HAQ (Health Assessment Questionnaire), evaluates daily life status in RA patients, where higher scores indicate lower health status (9). FJS-12, introduced by Behrend et al. in 2012 (10), assesses joint awareness in patients with prosthetic knees; higher scores indicate better knee proprioception, making it widely used for evaluating post-TKA recovery and quality of life (11).

### 2.6 Statistical analysis

Data were analyzed using SPSS 26.0 (IBM, USA). The Shapiro–Wilk test assessed normality. Data following normal distribution were presented as mean ± standard deviation, with paired t-tests for within-group comparisons. Data with non-normal distribution were shown as median (interquartile range) and analyzed using the Mann–Whitney *U* test. *P* < 0.05 was considered statistically significant.

## 3 Results

### 3.1 General results

**Figure 1:**
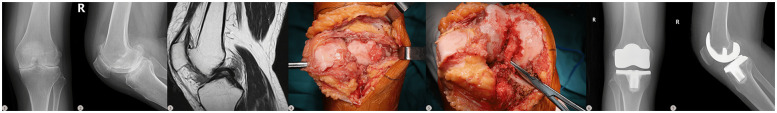
A 59-year-old female with a 13-year history of rheumatoid arthritis (RA). Preoperative imaging and intraoperative exploration revealed an intact and stable PCL, prompting the selection of a CR prosthesis for TKA. ①–②: the preoperative x-ray findings, ③: the preoperative MRI results, ④–⑤: the intraoperative exploration, ⑥–⑦: the postoperative follow-up x-ray results.

**Figure 2:**
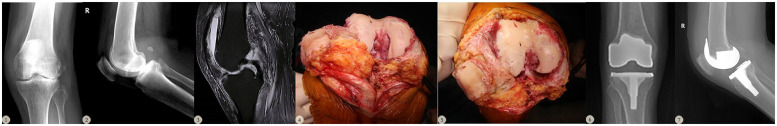
A 70-year-old female with a 31-year history of rheumatoid arthritis (RA). Preoperative imaging and intraoperative exploration revealed an incomplete and unstable PCL, prompting the selection of a PS prosthesis for TKA. ①–②: the preoperative x-ray findings, ③: the preoperative MRI results, ④–⑤: the intraoperative exploration, ⑥–⑦: the postoperative follow-up x-ray results.

A total of 74 patients were included, with 39 in the CR group (26 females and 13 males) and 35 in the PS group (23 females and 12 males). Baseline characteristics were comparable between the two groups, with no statistically significant differences except for the operation time. Detailed information is shown in Table 1. All patients successfully completed the surgery without significant postoperative complications. Representative case images are shown in Figures 1, 2.

**Table 1:** Characteristics of included patients.

Variables	CR group (*n* = 39)	PS group (*n* = 35)
Age	61.82 ± 9.74	65.14 ± 8.49
Gender		
Male	13	12
Female	26	23
BMI	21.91 ± 3.32	22.95 ± 3.76
Disease Duration (years)	14.69 ± 5.8	15.51 ± 4.91
Surgical Time (min)	76.41 ± 12.38	84.46 ± 14.14*
Blood Loss (ml)	59.1 ± 28.24	61.0 ± 34.00
Hospital Stay (days)	9.64 ± 2.35	10.37 ± 2.29

* *P* < 0.05.

### 3.2 Follow-up results

The study results showed that the preoperative VAS score for the PS group was 7.21 ± 0.69, and for the CR group, it was 7.51 ± 0.61. The preoperative AKSS Knee Score was 50 ± 6.50 in the PS group and 49.28 ± 10.36 in the CR group, while the preoperative AKSS Function Score was 28.14 ± 6.54 in the PS group and 30.77 ± 7.12 in the CR group. The preoperative HAQ Score was 24.77 ± 5.23 in the PS group and 23.87 ± 6.38 in the CR group, shown in Table 2.

**Table 2:** Preoperative outcome measures for included patients.

Outcome measures	CR group (*n* = 39)	PS group (*n* = 35)
VAS score	7.51 ± 0.61	7.21 ± 0.69
AKSS knee score	49.28 ± 10.36	50 ± 6.50
AKSS function score	30.77 ± 7.12	28.14 ± 6.54
HAQ score	23.87 ± 6.38	24.77 ± 5.23

**Figure 3:**
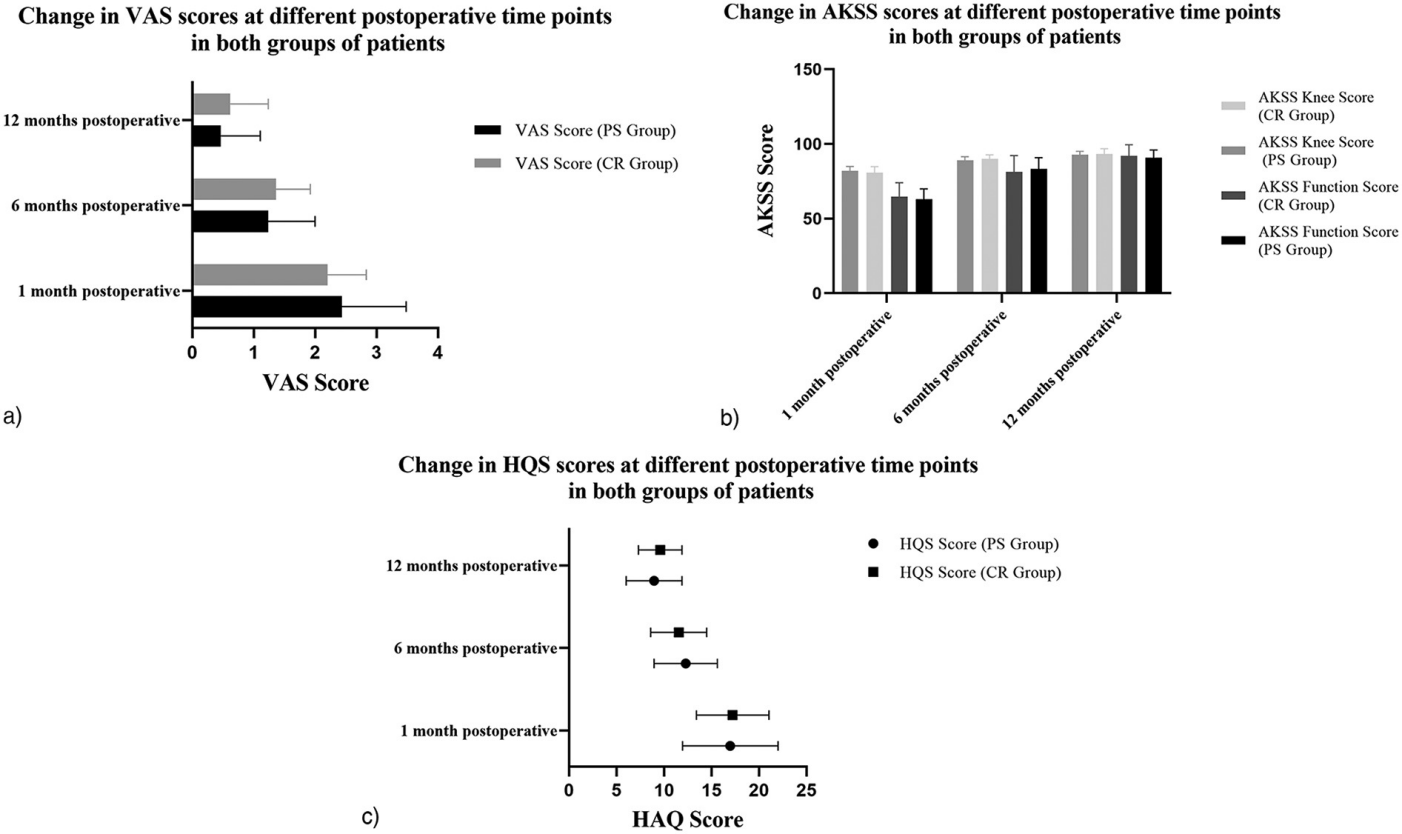
Changes in outcome measures at different time points after surgery in Two patient groups (**a**: VAS score, **b**: AKSS score, **c**: HAQ score).

At 1 month postoperatively, the VAS score was 2.20 ± 0.63 in the PS group and 2.44 ± 1.05 in the CR group. At 6 months, the VAS score decreased to 1.36 ± 0.56 in the PS group and 1.23 ± 0.77 in the CR group. By 12 months, the VAS score further decreased to 0.61 ± 0.62 in the PS group and 0.46 ± 0.65 in the CR group. At 1 month postoperatively, the AKSS Knee Score was 81.89 ± 3.11 and the Function Score was 63.00 ± 6.99 in the PS group, while in the CR group, the Knee Score was 80.69 ± 4.18 and the Function Score was 64.87 ± 9.14. At 6 months, the AKSS Knee Score in the PS group increased to 89.09 ± 2.29, with a Function Score of 83.14 ± 7.68; in the CR group, the Knee Score was 90.03 ± 2.67 and the Function Score was 81.15 ± 11.09. By 12 months, the AKSS Knee Score reached 92.63 ± 2.46 in the PS group with a Function Score of 90.86 ± 5.07, while in the CR group, the Knee Score was 93.26 ± 3.59 and the Function Score was 92.05 ± 7.41.For the HAQ Score, the PS group recorded 17.23 ± 3.82 at 1 month, 11.54 ± 2.9 at 6 months, and 9.6 ± 2.29 at 12 months postoperatively. In the CR group, the HAQ Score was 16.97 ± 5.03 at 1 month, 12.28 ± 3.34 at 6 months, and 8.95 ± 2.92 at 12 months. For the FJS-12 Score, the PS group had 31.97 ± 5.51 at 1 month, 43.57 ± 7.91 at 6 months, and 80.51 ± 5.79 at 12 months; the CR group had 32.03 ± 6.49 at 1 month, 50.93 ± 7.32 at 6 months, and 84.03 ± 4.20 at 12 months, shown in Figure 3.

Postoperative VAS and HAQ scores in both groups were significantly lower than preoperative scores, while AKSS scores were higher, with statistically significant differences (*P* < 0.05). Between-group comparisons showed no statistically significant differences in VAS, AKSS, or HAQ scores at any postoperative time points, shown in Tables 3–6. However, at 12 months postoperatively, the CR group had significantly higher FJS-12 scores compared to the PS group, shown in Table 7.

**Table 3:** Comparison of AKSS knee score after surgery between Two patient groups.

Group	*n*	1 Month postoperative	6 Months postoperative	12 Months postoperative
PS Group	35	81.89 ± 3.11	89.09 ± 2.29	92.63 ± 2.46
CR Group	39	80.69 ± 4.18	90.03 ± 2.67	93.26 ± 3.59
*t*		1.38	−1.62	−0.87
*P*		0.17	0.11	0.39

**Table 4:** Comparison of AKSS function score after surgery between two patient groups.

Group	*n*	1 month postoperative	6 months postoperative	12 months postoperative
PS group	35	65 (60, 70)	80 (80, 90)	90 (90, 90)
CR group	39	65 (60, 70)	85 (70, 90)	90 (90, 100)
*Z*		0.89	0.21	0.97
*P*		0.38	0.84	0.33

**Table 5:** Comparison of VAS score after surgery between two patient groups.

Group	*n*	1 month postoperative	6 months postoperative	12 months postoperative
PS group	35	2 (2, 3)	1 (1, 2)	1 (0, 1)
CR group	39	2 (2, 3)	1 (1, 2)	1 (0, 1)
*Z*		0.79	0.99	1.23
*P*		0.43	0.32	0.22

**Table 6:** Comparison of HAQ score after surgery between two patient groups.

Group	*n*	1 month postoperative	6 months postoperative	12 months postoperative
PS group	35	17.23 ± 3.82	11.54 ± 2.95	9.60 ± 2.29
CR group	39	16.97 ± 5.03	12.28 ± 3.34	8.95 ± 2.92
*t*		0.24	1.01	1.06
*P*		0.81	0.32	0.29

**Table 7:** Comparison of FJS-12 score after surgery between two patient groups.

Group	*n*	1 month postoperative	6 months postoperative	12 months postoperative
PS group	35	31.97 ± 5.51	43.57 ± 7.91	80.51 ± 5.79
CR group	39	32.03 ± 6.49	50.93 ± 7.32	84.03 ± 4.20
*t*		0.04	4.16	3.01
*P*		0.97	0.001	0.004

## 4 Discussion

Total knee arthroplasty (TKA) is currently the primary surgical treatment for advanced rheumatoid arthritis (RA) patients, as it can effectively relieve knee pain, improve mobility, and significantly enhance quality of life (12). However, there is still no consensus on whether to preserve the posterior cruciate ligament (PCL) in RA patients undergoing TKA. Some researchers argue that due to the inflammatory nature of RA, the functionality of the PCL may not be reliable as the disease progresses, and using a posterior cruciate ligament-retaining (CR) prosthesis could lead to postoperative posterior instability and increase the risk of revision surgery (13). Other studies, however, have demonstrated that the long-term survival rates of CR prostheses in RA patients are comparable to those of posterior-stabilized (PS) prostheses, with a low incidence of posterior instability (14). Since RA is characterized by bone destruction, RA patients undergoing TKA tend to be younger on average than osteoarthritis patients (15). For relatively younger RA patients, preserving more native soft tissue and bone mass may better maintain joint function postoperatively (16). Designed for bone preservation and low constraint, CR prostheses are considered more suitable than PS prostheses for RA patients with osteoporosis and higher activity demands (17).

Using a CR prosthesis in TKA can simplify the intercondylar bone resection, reduce operation time and intraoperative blood loss, and require less release of posterior soft tissue (16, 17). Long-term follow-up studies by Ricardo et al. (18) found no significant differences in pain scores between CR and PS groups, with similar rates of residual pain and swelling at the final follow-up. Another randomized study by Clark et al. (19) of 143 patients over more than two years showed no significant differences in functional scores or range of motion between the two prostheses. Similarly, long-term studies by Lauren et al. (20) and Ricardo et al. (18) over 10 years revealed similar AKSS and functional scores in both groups. In a large cohort study of 11,606 knee replacements, Rand et al. (17) concluded that posterior cruciate ligament-retaining prostheses had a long lifespan in elderly female patients with inflammatory arthritis. Our study corroborates these findings, with one-year follow-up showing significant improvements in AKSS, VAS, and HAQ score, in both CR and PS groups compared to preoperative levels, with no significant differences in scores between groups. No complications such as posterior instability, prosthesis loosening, infection, or fractures occurred during follow-up.

Compared to PS prostheses, CR prostheses retain the PCL, which can support femoral rollback mechanics, potentially providing a greater range of flexion postoperatively (21). Additionally, retaining the PCL can enhance joint mobility postoperatively (22). Hina et al. (23) found that in patients with preoperative varus deformity, 60% of those who received CR prostheses retained similar kinematics pre- and postoperatively, whereas only 25% of those with PS prostheses did, demonstrating that the PCL plays a crucial role in coronal knee stability postoperatively, aligning more closely with normal knee kinematics. An anatomical study by Kennedy et al. (24) on cadaver knee specimens also showed that the PCL's anterolateral and posteromedial bundles limit posterior tibial translation during knee flexion. Comparative studies on PS and CR prosthesis designs (25–27) indicate similar outcomes in mobility, aseptic loosening, polyethylene wear, and stability; however, Conditt et al. (28) reported that the cam mechanism in PS prostheses cannot fully replicate the function of the PCL, particularly in high-demand activities such as deep flexion, squatting, and kneeling. Thus, CR prostheses with PCL retention may offer better mobility and satisfaction in certain movements postoperatively compared to PS prostheses without PCL retention (8). FJS-12 scores showed no significant difference between groups at 1 month postoperatively, but the CR group exhibited better joint awareness than the PS group at 6 and 12 months. This may be due to some damage to the peripheral proprioceptive receptors during intraoperative release of the PCL and that occurs in the early postoperative period when the tissues are in a period of repair. As soft tissue and proprioceptive receptors heal by 6 months, the CR prosthesis, with its superior biological compatibility, provides better subjective movement sensation, enhancing joint awareness (29).

Additionally, Liu et al. (26) suggested that RA patients may have moderate to severe flexion contracture or PCL dysfunction, potentially requiring conversion from a CR to a PS prosthesis during surgery. Our study excluded patients with severe preoperative knee varus/valgus deformity or flexion contracture >10°, and we carefully assessed PCL integrity through repeated physical exams and MRI evaluation, along with intraoperative testing and PCL preservation (30). As a result, none of the CR group patients required conversion to a PS prosthesis due to PCL rupture or dysfunction intraoperatively.Our findings indicate that both CR and PS prostheses provide good postoperative outcomes for RA patients undergoing TKA. However, compared to PS prostheses, CR prostheses may offer better knee proprioception in the early postoperative period. We recommend careful evaluation of PCL integrity before and during surgery; RA patients with PCL damage should consider PS prostheses, while CR prostheses are more suitable for patients with an intact PCL and high functional demands for joint mobility postoperatively.

## Data Availability

The original contributions presented in the study are included in the article/Supplementary Material, further inquiries can be directed to the corresponding author.
